# Investigation of the Spatial Structure of Flufenamic Acid in Supercritical Carbon Dioxide Media via 2D NOESY

**DOI:** 10.3390/ma16041524

**Published:** 2023-02-11

**Authors:** Ilya A. Khodov, Konstantin V. Belov, Michael A. Krestyaninov, Alexey A. Dyshin, Michael G. Kiselev

**Affiliations:** G.A. Krestov Institute of Solution Chemistry of the Russian Academy of Sciences, Ivanovo 153045, Russia

**Keywords:** supercritical fluids, high-pressure NMR, spatial structure, micronization, fenamates, solubility

## Abstract

The search for new forms of already known drug compounds is an urgent problem of high relevance as more potent drugs with fewer side effects are needed. The trifluoromethyl group in flufenamic acid renders its chemical structure differently from other fenamates. This modification is responsible for a large number of conformational polymorphs. Therefore, flufenamic acid is a promising structural modification of well-known drug molecules. An effective approach in this field is micronization, employing “green” supercritical fluid technologies. This research raises some key questions to be answered on how to control polymorphic forms during the micronization of drug compounds. The results presented in this work demonstrate the ability of two-dimensional nuclear Overhauser effect spectroscopy to determine conformational preferences of small molecular weight drug compounds in solutions and fluids, which can be used to predict the polymorphic form during the micronization. Quantitative analysis was carried out to identify the conformational preferences of flufenamic acid molecules in dimethyl sulfoxide-d6 medium at 25 °C and 0.1 MPa, and in mixed solvent medium containing supercritical carbon dioxide at 45 °C and 9 MPa. The data presented allows predictions of the flufenamic acid conformational preferences of poorly soluble drug compounds to obtain new micronized forms.

## 1. Introduction

Flufenamic acid (2-{[3-(trifluoromethyl)phenyl]amino}benzoic acid) (FFA) is a representative member of the fenamates pharmacological group. Until recently, it was actively used in medical practice as an analgesic with anti-inflammatory and antipyretic actions [1].

FFA occupies a special place among fenamates due to its specific chemical structure and variety of polymorphic forms. It is known to comprise eight polymorphic forms, which makes is rather unique among other small molecilar drug compounds [2,3,4,5]. The decisive feature of the FFA structure responsible for the considerable interest in drug design and development [6] is the presence of a trifluoromethyl group. Compounds with fluorine-containing substituents are known to feature promising chemical and biological properties [7,8,9] because such groups often improve drug pharmacokinetics and bioavailability [10]. Active pharmaceutical ingredients (APIs) synthesized by Dorsey and co-authors [11] as derivatives of fluorine-containing N-substituted benzamides have been shown to exhibit anti-inflammatory activity, which is determined, among other factors, by the trifluoromethyl group position in their structure [12,13].

Although FFA has been proven to be effective in the treatment of rheumatoid arthritis, osteoarthritis and other diseases accompanied by inflammation [14], the application of FFA is restricted in the Russian Federation and the United States of America because the drug causes a number of side effects [15]. FFA, as a compound of class II, according to the biopharmaceutical classification system (BCS) [16], is poorly soluble in water. There is considerable interest to increase the solubility and, as a result, the bioavailability of FFA [17] in order to minimize its side effects, optimize production of its micronized forms and bring it back to the pharmaceutical market.

One way to improve drug solubility that has recently gained popularity is micronization, based on “green” supercritical fluid (SCF) technologies. SCF processes often employ carbon dioxide (SC-CO_2_) as the inert solvent because the CO_2_ transition to the fluid state occurs at relatively low pressure and temperature values (31.1 °C and 7.38 MPa), and the solvent can be easily removed through system decompression. Furthermore, the environmentally friendly character of this “green” solvent is the result of its recyclability. In 2004, the authors of work [18] claimed SCF technologies to be the future of the pharmaceutical industry. Today, SC-CO_2_-based methods, such as Rapid Expansion of Supercritical Solutions (RESS) [19,20], Supercritical Anti Solvent (SAS) [21,22], Particles from Gas Saturated Solutions (PGSS) [23,24], etc., are frequently used to prepare micronized forms of drug compounds with improved properties [25]. The process of obtaining micronized forms of drug compounds based on SCF technologies must also include the control of crystals polymorphic forms. Several studies [26,27,28] have reported that micronization of stearic acid, ibuprofen, phenylbutazone, etc., can be accompanied by changes in polymorphic forms.

Controlling the composition of ultra-fine drug forms preparation is a separate complicated research problem because their polymorphic composition of micronized forms can only be determined by a few methods. The most common method of solving this problem is fast differential scanning calorimetry [29]. Sometimes, X-ray diffraction analysis (XRD) and powder diffraction analysis are also applied [30]. Oparin et al. [31] showed a correlation between the conformers of mefenamic acid in the SC-CO_2_ solution and the polymorphic composition in the solid state [31]. Therefore, information about the conformational preferences of drug molecules in solution at supercritical parameters of the state of CO_2_ can serve as the “fingerprint” for probable polymorphic transitions. Since FFA is a fenamate and has the biggest number of conformational polymorphs (at least eight forms are known), an urgent problem for SCF-based micronization is the identification of its conformational preferences in the SC-CO_2_ medium.

The XRD and Differential Scanning Calorimetry (DSC) methods proved quite effective in revealing the mechanisms and seeking the regularities in formations of polymorphs in solid–solid or solid–liquid transitions. For example, monitoring polymorphic phase transitions in flufenamic acid by XRD and DSC has shown the possibility of inhibiting the formation of certain polymorphic forms through fixing molecules of FFA in a polymer in a certain configuration/conformation [32]. On the other hand, Nechipadappu, Joshi and their research groups showed [1,33], using the same experimental methods, that fixing the FFA molecules in a given configuration is possible upon the formation of co-crystals. In some studies, transformation of polymorphic forms caused by changes in the solution’s volume have been observed [31,34]. Nuclear Magnetic Resonance (NMR) and, in particular, nuclear Overhauser effect (NOE) spectroscopy are promising tools for experimental studies of such systems, including systems with supercritical parameters of the solvent state. NOESY approaches at supercritical parameters have not yet been well developed, and hence, results obtained in the present research should be useful. This study of flufenamic acid and its conformations state in the solution volume will provide more information to the DSC and XRD data present in the literature and improve our understanding of the formation of polymorphic forms at the molecular level. Notably, classical methods of DSC are poorly applicable for fine particles and so fast scanning calorimetry is used, which has limited capabilities for this kind of problem [29].

The nuclear Overhauser effect spectroscopy (2D NOESY) technique is a powerful tool for chemical structure elucidation of small drug-like molecules by NMR. In the last few years [35,36,37,38,39,40,41,42,43,44,45,46,47,48], this technique has been extensively used to study the structure and conformational preference of small molecules in supercritical media. The value of the cross-relax rate between proton spins in a small molecule of FFA is proportional to the sixth power of the distance between the corresponding protons. In this paper, we propose novel data based on quantum mechanical considerations and NOESY distances for estimating preference conformers of weak soluble FFA in SC-CO2 media. The specific feature of FFA is its poor solubility in SC-CO_2_ (<0.3 g × L^−1^) at the ρCO_2_ = 285.00 g × L^−1^ [49]. In order to improve the FFA solubility [50], we added 2 mol% of the pharmaceutically relevant cosolvent DMSO [51]. The use of such relatively small amounts of DMSO-d_6_ ensured that the FFA concentration was suitable for NMR analysis. The state parameters (45 °C and 9 MPa) for handling the mixed solvent were selected in accordance to the literature data [52,53] and were prompted by the phase behavior of the SC-CO_2_/DMSO mixture, which is sometimes employed in micronization processes of drug compounds, such as the Depressurization of an Expanded Liquid Organic Solution (DELOS) [54].

The aim of this research is to determine the flufenamic acid conformational state in bulk solution to improve our understanding of possible mechanisms of crystalline polymorphic forms formation.

This work presents the results of the conformational analysis of FFA in DMSO-d_6_ (25 °C and 0.1 MPa) and a mixed solvent containing SC-CO_2_+DMSO-d_6_ (45 °C and 9 MPa). The results were obtained using the nuclear Overhauser effect spectroscopy method.

## 2. Materials and Methods

### 2.1. Experimental Section

In this work, we used flufenamic acid 99.99 wt% (CAS No. 530-78-9) and anhydrous deuterated dimethyl sulfoxide (DMSO-d_6_) (CAS No. 2206-27-1) produced by Sigma Aldrich and carbon dioxide (ultrahigh purity CO_2_), GOST 8050–85 (CO_2_ = 99.97%, H_2_O < 0.001%) produced by Chistye Gazy Plus, OOO (Novosibirsk). The saturated FFA solution in DMSO-d_6_ was prepared in a standard NMR tube without additional purification of the sample. The FFA sample was prepared based on the literature data on the solubility in DMSO-d_6_ [55].

The sample was prepared in several steps. A certain weight of FFA (540 mg) was dissolved in 1 mL DMSO-d_6_ in a standard glass NMR tube until a stable solid phase was formed at 45 °C. The temperature of the sample was maintained using a laboratory air thermostat. After that, 86 μL of the liquid phase was taken and immediately put into a special NMR tube made of sapphire single crystal. Then, the cell containing FFA in DMSO-d_6_ was filled with carbon dioxide from the gas cylinder, until the pressure limit of 9 MPa was achieved. Since FFA dissolves much better in DMSO-d_6_ than in the mix SC-CO_2_ + DMSO-d_6_, a small amount of the solid phase was formed, which was a necessary condition for the experiment. At the same time, an addition of 2 mol % DMSO-d_6_ at the mentioned parameters of state of the system provided the necessary concentration of the material in bulk solution in the NMR coil region. After the sealing and setting of the necessary parameters, the cell with the sample was put into the NMR probehead, where the temperature was stabilized using the spectrometer’s equipment (BVT-2000 and BCU-05, Bruker Biospin, Karlsruhe, Baden-Württemberg, Germany). NMR spectra were only recorded after the thermodynamic equilibrium in the system had been achieved; this was checked by stabilization of the spectral characteristics of the signals (chemical shift, peak width, integral intensity) in the test ^1^H spectra.

**Figure 1:**
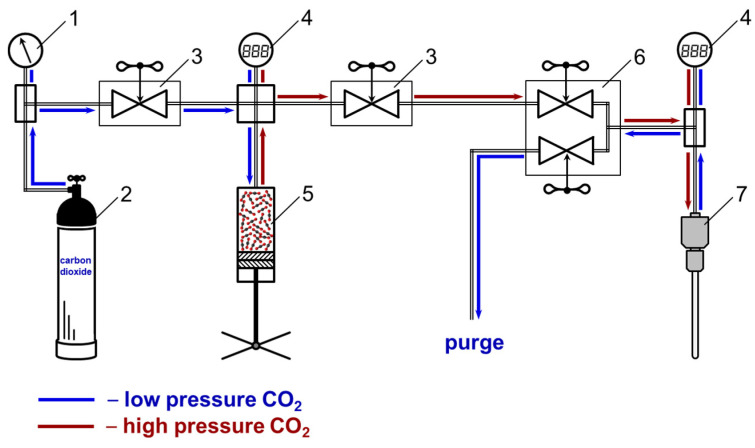
Scheme of the apparatus to produce and maintain pressure for NMR measurements, where 1 is a pressure gauge, 2 is a cylinder with carbon dioxide, 3 and 6 are taper seal valves, 4 are electronic pressure transmitters, 5 is a manually operated syringe pump, and 7 is a high-pressure NMR cell.

The NMR experiments in a mixed SC-CO_2_-based solvent were carried out using a real-time system for producing and maintaining high pressure (see Figure 1) on our homemade setup “Fluid-Spectrum” https://ckp-rf.ru/catalog/usu/503933 (accessed on 20 December 2022) at the G.A. Krestov Institute of Solution Chemistry of the Russian Academy of Sciences. The device comprised of a system of taper seal valves (Figure 1, pos. 3 and 6) and a stainless steel high-pressure capillary connected to a high-pressure NMR cell (Daedalus Innovations LLC, Aston, PA, USA) (Figure 1, pos. 7), which allows carbon dioxide to be added from a cylinder (Figure 1, pos. 2). The pressure was regulated with a hand press (HiP Co.) (Figure 1, pos. 5) and controlled with a pressure gauge (Figure 1, pos. 1) and electronic pressure transmitters (Gems™ Sensors&Controls, Inc., Plainville, CT, USA) (Figure 1, pos. 4). The accuracy of pressure maintenance was ±0.05 MPa. The improvement procedure of the commercial NMR cell was previously described in work [39].

This setup was linked to a Bruker Avance III 500 spectrometer (Bruker Biospin, Karlsruhe, Baden-Württemberg, Germany) and a high pressure NMR cell (Daedalus Innovations LLC, Aston, PA, USA) recording the NMR spectra at given parameters of state exceeding the critical point for CO_2_ (45 °C and 9 MPa). At such pressure and temperature values, the SC-CO_2_ + DMSO-d_6_ mixture can be in a subcritical state [52,56,57].

To prepare the sample for the NMR experiments, we placed a fixed amount of a saturated FFA solution in DMSO-d_6_ (86 µL) into a sapphire cell and then filled the remaining volume of the cell with carbon dioxide under a pressure of 9 MPa. The prepared samples were used to record 1D (^1^H, ^13^C) and 2D (^1^H-^13^C HSQC, ^1^H-^13^C HMBC, ^1^H-^1^H TOCSY, ^1^H-^1^H NOESY) NMR spectra of FFA in DMSO-d_6_ and SC-CO_2_+DMSO-d_6_. The NMR frequency for ^1^H nuclei was 500.17 MHz and for ^13^C—125.77 MHz. The temperature was kept constant with an accuracy of ± 0.10°C using temperature control (BVT-2000, Bruker Biospin, Karlsruhe, Baden-Württemberg, Germany) and cooling (BCU-05, Bruker Biospin, Karlsruhe, Baden-Württemberg, Germany) units.

The ^1^H NMR spectrum (see Figure S1) was recorded using a sweep width of 20 ppm with 512 scans [58]. The ^13^C NMR spectrum (see Figure S2) was acquired with a sweep width of 276 ppm, accumulating 4096 scans. The relaxation delay was 2 s. Tetramethylsilane (TMS) signal (δ_TMS_ = 0 ppm) was used as the standard to calibrate the chemical shifts in the ^1^H NMR spectra.

The inter-proton distances of flufenamic acid molecules are determined from the value cross-peaks assigned by proton chemical shifts in two-dimensional NMR spectra (HSQC and HMBC). These chemical shifts obtained by HSQC and HMBC for NOESY experiments are used as a source of information in the structure determination of molecules. Therefore, obtaining HSQC and HMBC data is a necessary step to determine the conformational preferences of drug compounds’ small molecules. To record the ^1^H-^13^C HSQC (heteronuclear single quantum coherence spectroscopy) spectrum [59,60,61] (see Figure S3) we chose a spectral window of 20 ppm × 276 ppm, with 256 data points on the F1 axis and 1024 points on the F2 axis; the number of scans was 16. The ^1^H-^13^C HMBC (Heteronuclear multiple-bond correlation spectroscopy) spectra [62] (see Figure S4) were recorded within the same frequency range, with 256 data points in the F1 direction and 4096 in the F2 dimension; the number of scans was 120. The homonuclear ^1^H-^1^H TOCSY (Total Correlation Spectroscopy) spectra [58,63] (see Figures S5–S7) were recorded applying three different mixing times (20 ms, 60 ms and 100 ms), within a spectral window of 20 ppm × 20 ppm and 16 scans.

The nuclear Overhauser effect spectra were recorded [64,65,66] in a spectral window of 20 ppm × 20 ppm using 16 (DMSO-d_6_) or 8 (SC-CO_2_) scans. Mixing times in the NOESY experiments in DMSO-d_6_ were 0.05, 0.10, 0.15, 0.20, 0.25, 0.30, 0.35, 0.40, 0.45, 0.50, 0.55, 0.60, 0.65, 0.70, 0.75 and 0.80 s. For SC-CO_2_+DMSO-d_6_, mixing times were 0.10, 0.20, 0.30, 0.40, 0.50, 0.60, 0.70, 0.80 and 0.90 s.

The chemical shifts of the signals in the ^1^H and ^13^C spectra and the assignment of the cross-peaks in the ^1^H-^13^C HSQC, ^1^H-^13^C HMBC and ^1^H-^1^H TOCSY spectra are shown in the table (Table S1).

### 2.2. Quantum-Chemical Calculations

The quantum-chemical calculations of the FFA conformer structure geometry and energy were performed using the Gaussian 09 software package [67]. The first step consisted of searching for probable FFA conformers by analyzing the potential energy surface scans using the PM3 (Parametrical Method 3) semiempirical method [68] to obtain preliminary results. The next step included optimization of the geometry and vibrational frequencies based on the density functional theory (DFT) with the Austin–Frisch–Petersson APFD functional [69] and 6–311++g(2d,p) basis set [70,71]. The APFD functional was chosen because of its applicability [69,72] to working with organic drug compounds of small molecules, having a cyclic fragment in the structure. The calculations of the obtained minima were confirmed by the absence of imaginary vibrational frequencies during the conformational search. The parameters of the intramolecular hydrogen bonds were calculated and the energy barriers of the transitions between the conformers were determined for the four most stable conformers (A, B, C and D).

## 3. Results and Discussions

### 3.1. Analysis of the FFA Structure

**Figure 2:**
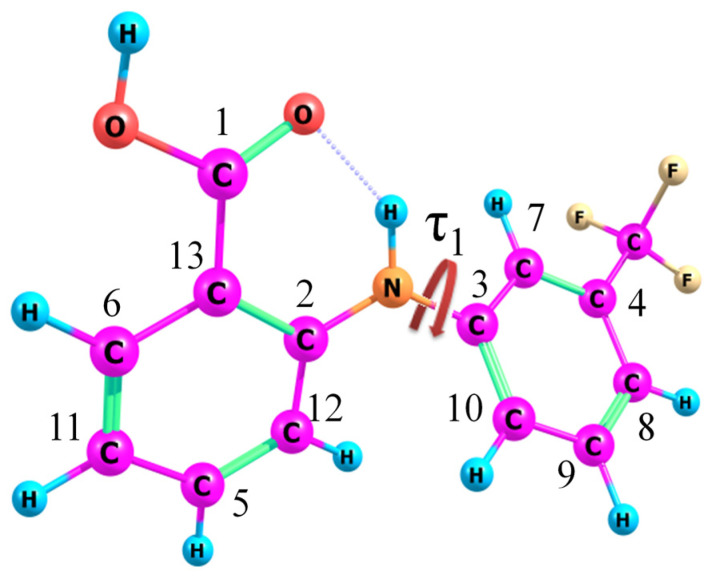
Structural formula of FFA. Atom numbering is used to label the signals and cross-peaks in the NMR spectra and to denote the dihedral angles. The dihedral angle τ_1_[C_2_-N-C_3_-C_7_] is shown by the red arrow.

Flufenamic acid (N-(3-Trifluoromethyl phenyl)-anthranilic acid) is a biologically active compound of the fenamate pharmacological group belonging to a large class of non-steroidal anti-inflammatory drugs (NSAIDs). The FFA chemical structure contains a mono-carboxylate nucleus of diphenylamine substituted for a trifluoromethyl radical (see Figure 2). FFA conformers are known [2,73,74] to have different values of the dihedral angle τ_1_[C_2_-N-C_3_-C_7_] related to the rotation of the benzene rings of the diphenylamine fragment relative to each other.

The FFA conformation associated with the orientation of the diphenylamine fragment benzene rings relative to each other (τ_1_[C_2_-N-C_3_-C_7_]) determines which polymorphic form is populated out of the eight polymorphic structures described so far [2,3,4,5]. The authors of the study described in [3] assumed that FFA may assume one more form (IX), but the geometric parameters of its molecular structure are unknown yet. The values of the dihedral angle τ_1_ for all the known FFA structures realized in various polymorphic forms are given in SI (Table S2).

As described above, to find new conformational FFA polymorphs by micronization, we identified the conformer populations based on quantum-chemical calculations and NMR experiments.

**Figure 3:**
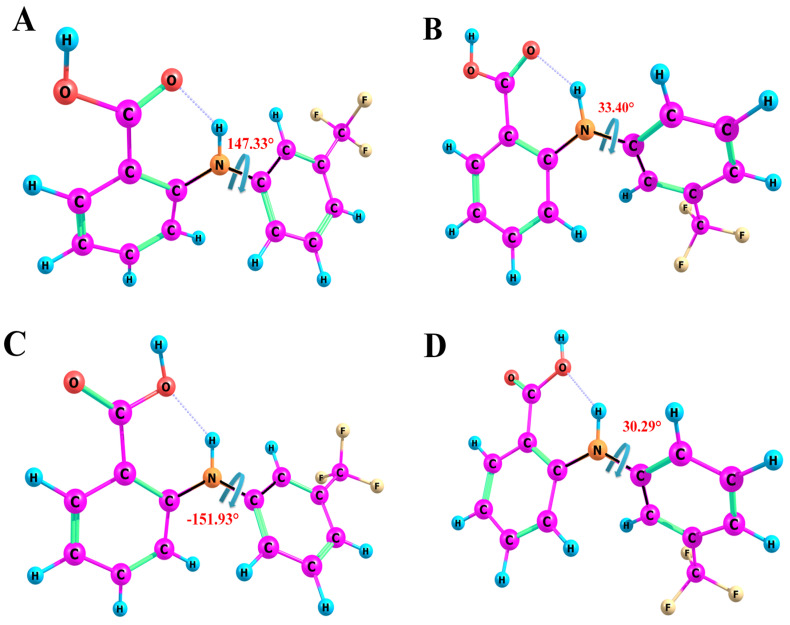
Structures of FFA molecule conformers with dihedral angles τ_1_[C_2_-N-C_3_-C_7_] (black highlights). The four most stable conformers of FFA (**A**–**D**) with an intramolecular H-bond, which are considered later in the NMR data analysis. Conformers A/C and B/D are distinguished by the rotation of the trifluoromethyl-substituted moiety relative to the remaining part of the molecule. Conformers A/B and C/D are distinguished by the rotation of the COOH fragment.

The quantum-chemical calculations yielded the four most probable FFA conformers (A, B, C and D) depending on the values of the τ_1_ angle [C_2_-N-C_3_-C_7_] (A/C and B/D). We did not make any calculations that would take into account the medium by applying a polarizable continuum model (PCM) because this would significantly limit the results of the conformational search [75] and could lead to the wrong interpretation of the NOESY results. The structures of the probable conformers are given in Figure 3.

As Figure 3 shows, the trifluoromethyl fragment can occupy different positions: it can be codirectional to the carboxyl group of the anthranilic fragment (conformers A and C) and contradirectional (B and D). In this case, the value of the dihedral angle τ_1_[C_2_-N-C_3_-C_7_] for conformers A and C, on the one hand, and B and D, on the other hand, changes from −30° to −150°, respectively. The conformers shown in Figure 3 have different orientations of the carboxyl fragments, but, as reported in the literature [3], this type of molecular lability does not directly affect the formation of FFA polymorphic forms.

### 3.2. Quantum-Chemical Calculations

Quantum-chemical calculations yielded 16 low-energy structures. The parameters of the four most stable conformers with an intramolecular H-bond, which are considered later in the NMR data analysis, are listed in Table 1. Some conformers are stabilized by the π–π conjugation interaction of the aromatic rings through the electron pairs of the nitrogen atom and the benzene rings, which make the C_2_-N(H)-C_3_ fragment configuration planar. Rotation of the hydroxyl fragment within the carboxyl group and of the aromatic ring with a carboxyl group produced conformers with higher energies. Therefore, they were excluded from further analysis.

**Figure 4:**
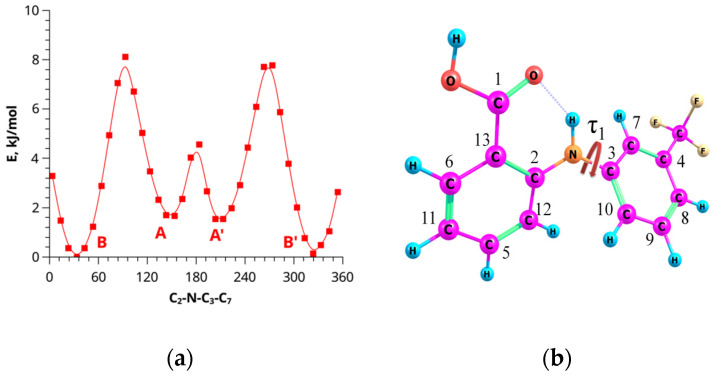
Barriers of intramolecular rotation related A(C) and B(D) (**a**) to the C2-N-C3-C7 angle (**b**) in the FFA molecule by quantum chemical calculations.

According to the quantum-chemical calculations, conformer B has the lowest energy and it was chosen as the reference structure for comparing the total energies of the other conformers. Conformers B and A are distinguished by the rotation of the trifluoromethyl-substituted moiety relative to the remaining part of the molecule (angle τ_1_, C_2_-N-C_3_-C_7_). The relative energies and the barrier between these two forms are relatively small (Figure 4a). The resulting values of the intramolecular H-bonds stabilization energy in these two conformers obtained by the NBO method are nearly the same (55.23 kJ/mol for B and 54.73 for A; see Table 1). Conformers A’ and B’, shown in Figure 4a, are mirror symmetry conformers of forms A and B, and possess the same geometric (the angle τ_1_ = 33.40° and 147.33°, respectively) and energy parameters. These structures are indistinguishable in the NMR experiments. The transition barriers A → A’ (~3 kJ/mol) and B → B’ (~3 kJ/mol) are lower than A → B (~8 kJ/mol) due to the steric factors caused by the substituents’ influence on the molecular geometry of the aromatic rings.

Conformers C and D differ in the O=C_1_-C_13_-C_6_ (τ_2_) angle, i.e., due to the rotation of the carboxyl groups relative to the benzene ring. They have higher energies (16.96 and 17.77 kJ/mol) and lower stabilization energy of the intramolecular H-bonds (27.41 and 27.28 kJ/mol). At the same time, the barriers for A/B → C/D transitions are substantially higher, about 50 kJ/mol (Figure S8), which is due to the O···H–NH-bond breakage. For this reason we only considered two main conformer groups where the variation of the angle τ_1_ was within 1–2°.

### 3.3. NOESY Spectroscopy Results

**Figure 5:**
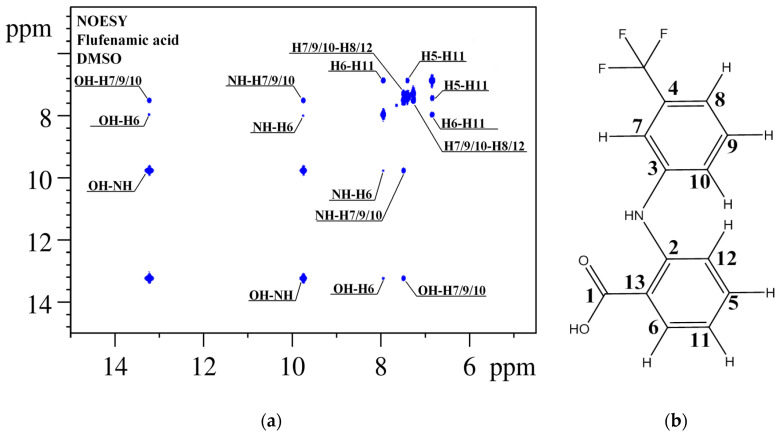
^1^H-^1^H NOESY NMR spectrum (**a**) of FFA (**b**) in DMSO-d_6_. The observed cross-peaks correspond to the groups of ^1^H-^1^H atoms located at a distance of up to 5 Å from each other and are numbered according to the structure shown in Figure 2.

For a quantitative analysis of the ^1^H-^1^H NOESY spectra, we identified signals in the 1D NMR (^1^H and ^13^C) spectra of the FFA molecule (see Figures S1 and S2) using 2D NMR (^1^H-^13^C HSQC, ^1^H-^13^C HMBC and ^1^H-^1^H TOCSY) methods (see Figures S3–S7). The assignment of the ^1^H NMR signals was used to interpret the cross-peaks observed in the NOESY spectra for FFA (see Figure 5).

To find the FFA conformer populations, we experimentally determined the values of the NH-H7 distance based on the NOESY spectroscopy data. The approach used to determine the distances is based on the interconnection between the cross-relaxation rate and the internuclear distance according to proportional 1.

(1) $$\sigma_{ij}=1/r_{ij}{}^{6}$$

where *σ_ij_* is the rate of cross-relaxation between the *i*th and *j*th atoms, and *r_ij_* is the distance in Å between the *i*th and *j*th atoms.

**Figure 6:**
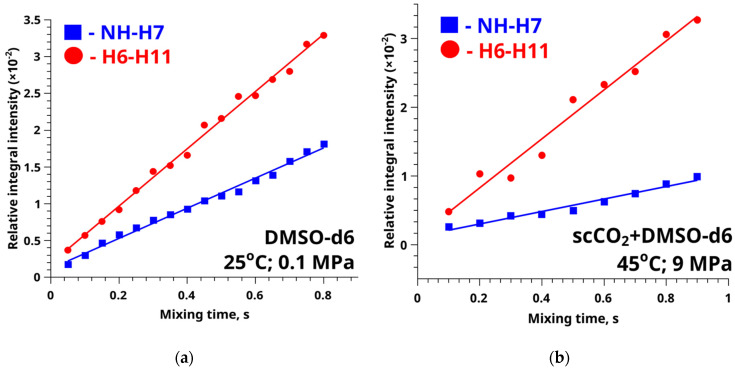
Graphs of averaged integral intensity vs. mixing time for the conformational (the red line) and reference (the blue line) distances obtained by analyzing the NOESY spectra of FFA-DMSO-d_6_ (**a**) and FFA-SC-CO_2_+DMSO-d_6_ at 45 °C and 9 MPa (**b**).

Lee and Krishna [76] showed that, in cases of fast conformational exchange, the resulting cross-relaxation rate is a weighted average of the values of the individual conformers (see Equation (2)). Therefore, the value of the distance obtained will be the weighted average of 1/*r*^6^. The cross-relaxation rate can be determined from the slope parameters of the approximating straight line of the dependence of the averaged integral cross-peak intensity in the NOESY spectrum (see Equation (3)) vs. “mixing time” (*I_ij_(τ_m_)*) (see Figure 6). The data are given in SI. (Tables S3 and S4).

(2) $$\sigma_{\exp}=\sum_{i}\sigma_{i}x_{i}$$

Here, *σ_exp_* is the resulting cross-relaxation rate, *σ_i_* is the cross-relaxation rate in the *i*th conformer, *x_i_* is the population of the *i*th conformer. The normalized cross-peak intensity is

(3) $$I_{ij}(\tau_{m})=1/2\left(1/n_{j}\left|a_{ij}(\tau_{m})/a_{ii}(\tau_{m})\right|+1/n_{i}\left|a_{ji}(\tau_{m})/a_{jj}(\tau_{m})\right|\right)$$

where *n_j_* and *n_i_* are the parameters indicating the number of protons in the group, *a_ij_* and *a_ji_* are the parameters determining the cross-peak integral in the 2D NOESY spectra, *a_ii_* and *a_jj_* are the parameters determining the integral of the diagonal signals in the 2D NOESY spectra.

The values of the cross-relaxation rates according to the NOESY data for the NH-H7 and H6-H11 groups of atoms in the FFA molecule in the DMSO-d_6_ medium were: (2.05 ± 0.05) × 10^−2^ s^−1^ and (3.89 ± 0.14) × 10^−2^ s^−1^, respectively. However, the experimental values of the cross-relaxation rates for these distances in the SC-CO_2_+DMSO-d_6_ medium at 45 °C and 9 MPa were: (9.08 ± 0.63) × 10^−3^ s^−1^ and (3.56 ± 0.22) × 10^−2^ s^−1^, respectively.

Using the isolated spin-pair approximation (ISPA) model [39,77,78] (see Equation (4)), we obtained the values of the NH-H7 distances for the FFA system in DMSO-d_6_ to be 2.75 ± 0.03 Å and 3.10 ± 0.07 Å for FFA in the SC-CO_2_ medium with a small addition of DMSO-d_6_:

(4) $$r_{\exp}=r_{0}\sqrt[{6}]{\sigma_{0}/\sigma_{\exp}}$$

where *r*_0_ is the reference distance obtained from experimental XRD analysis data, σ_0_ is the cross-relaxation rate for the reference distance, *σ*_exp_ is the cross-relaxation rate for the distance to be found, *r*_exp_ is the internuclear distance obtained from the NOESY experiment.

**Figure 7:**
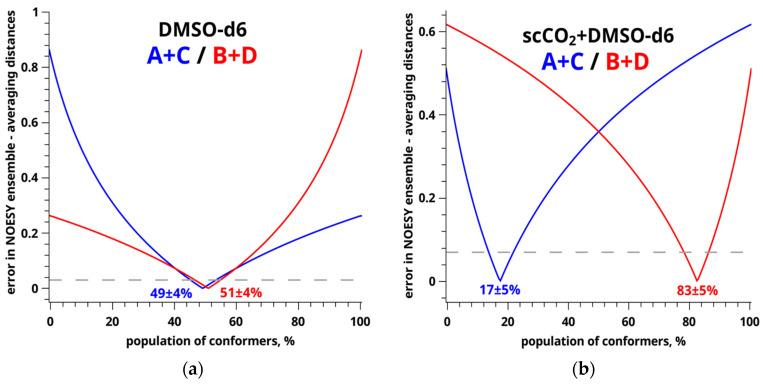
Graphs of the difference between the calculated and experimental distances vs. conformer populations, where fractions of A+C are blue lines; the fractions of B+D are red lines; dashed gray lines are the distance accuracy levels. The experimental data were obtained from the FFA NOESY spectra recorded in DMSO (**a**) and SC-CO_2_ + DMSO (**b**).

**Figure 8:**
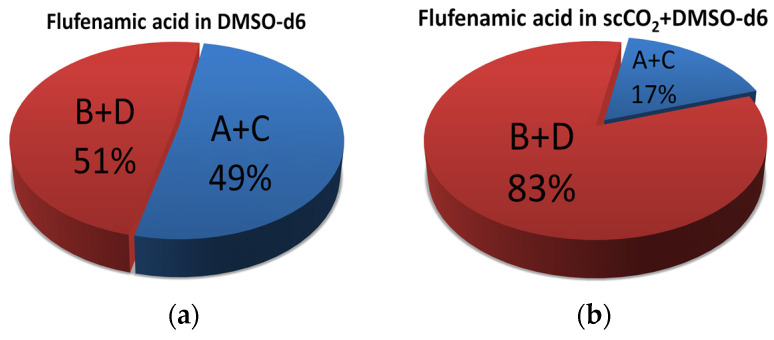
Distribution of conformers of FFA in DMSO (**a**) and SC-CO_2_+DMSO (**b**) calculated from the observed conformation-dependent distance NH-H7.

To determine the accuracy of conformer population estimation by a two-position exchange equation (see Equation (5)), we plotted a graph of the dependence of the difference between the calculated (see Table 2) and experimental values of the distance to be found on the conformer populations for FFA in DMSO-d_6_ and mixed SC-CO_2_+DMSO-d_6_ solvent (see Figure 7 and Figure 8) [37,39,79]. 

According to the graphs, the perpendicular line drawn from the points of the intersection of the dependence graph and the experimental error graph (the grey line) shows the error range.

(5) $$x_{1}=r_{1}^{6}(r_{2}^{6}-r_{\exp}^{6})/r_{\exp}^{6}(r_{2}^{6}-r_{1}^{6})\to x_{2}=1-x_{1}$$

Here, *r*_0_ is the reference distance obtained from the experimental XRD analysis data, σ_0_ is the experimental cross-relaxation rate of the reference distance, *σ*_exp_ is the cross-relaxation rate of the distance to be found, r_1_ and r_2_ are the internuclear distances of the conformer groups, *r*_exp_ is the internuclear distance obtained from the NOESY experiment data.

The minima on the graphs correspond to the populations of the A+C (the blue curve) and B+D (the red curve) conformers. For example, in the case of DMSO-d_6_, the populations of the A+C (49%) and B+D (51%) conformers are equal within the measurement accuracy (±4%), whereas the conformational preferences in the system with the mixed SC-CO_2_+DMSO-d_6_ solvent are significantly different: A+C (17%) and B+D (83%). The data obtained are shown in Figure 8.

## 4. Conclusions

In this work, we carried out a quantitative analysis of conformational preferences of flufenamic acid, a poorly soluble drug compound, in DMSO-d_6_ and a mixed solvent—SC-CO_2_+DMSO-d_6_. The populations of A+C and B+D conformers associated with the benzene ring rotation around the NH-C3 bond and differences in the dihedral angle τ_1_[C_2_-N-C_3_-C_7_] were 49/51 ± 4% in DMSO-d_6_ and 17/83 ± 5% in the mixed SC-CO_2_+DMSO-d_6_ solvent. The FFA conformational preferences identified using nuclear Overhauser effect spectroscopy (NOESY) agreed with the results of the quantum-chemical calculations. The predominant conformations of the FFA molecules were B+D regardless of the solvent used. However, the conformer populations were significantly different (by 32%). Although DMSO and SC-CO_2_ mixed systems were used in crystallization experiments, it is difficult to infer crystal nucleation and growth mechanisms in other solvent systems based on this result alone.

## Figures and Tables

**Table 1:** Dihedral angles, relative energies (ΔE), geometric parameters, stabilization energies of the second order (E^2^), and charge transfer (q) for intramolecular hydrogen bonds for four FFA conformers.

Conf.	C_2_-N-C_3_-C_7_, °	O=C_1_-C_13_-C_6_, °	R(NO), Å	R(HO), Å	(NHO),O	E^2^, kJ	q, e	ΔE, kJ/mol
B	33.40	−5.59	2.651	1.814	136.95	55.23	0.0276	0.00
A	147.33	5.31	2.650	1.816	163.68	54.73	0.0276	1.62
D	30.29	171.05	2.664	1.886	131.57	27.41	0.0095	16.96
C	−151.93	−170.77	2.663	1.886	131.37	27.28	0.0095	17.77

**Table 2:** Conformational and reference distances for FFA conformers according to quantum-chemical calculations.

Interproton Distances, Å	Conformers			
	A	B	C	D
NH-H7	2.51	3.58	2.46	3.58
H6-H11	2.45	2.47	2.45	2.48

## Data Availability

Not applicable.

## Supplementary Materials

The following supporting information can be downloaded at: https://www.mdpi.com/article/10.3390/ma16041524/s1, Figure S1: ^1^H NMR spectrum of FFA in DMSO-d_6_ was recorded on a Bruker Avance III spectrometer, 500 MHz, in the frequency range of 20 ppm; Figure S2: ^13^C NMR spectrum of FFA in DMSO-d_6_ was recorded on a Bruker Avance III spectrometer, 500 MHz, in the frequency range of 276 ppm; Figure S3: ^1^H-^13^C HSQC spectrum of FFA in DMSO-d_6_ was recorded on a Bruker Avance III spectrometer, 500 MHz, in the frequency range of 20 × 276 ppm; Figure S4: ^1^H-^13^C HMBC spectrum of FFA in DMSO-d_6_ was recorded on a Bruker Avance III spectrometer, 500 MHz, in the frequency range of 20 × 276 ppm; Figure S5: ^1^H-^1^H TOCSY spectrum (mixing time parameter—20 ms) of FFA in DMSO-d_6_ was recorded on a Bruker Avance III spectrometer, 500 MHz, in the frequency range of 20 × 20 ppm; Figure S6: ^1^H-^1^H TOCSY spectrum (mixing time parameter—60 ms) of FFA in DMSO-d_6_ was recorded on a Bruker Avance III spectrometer, 500 MHz, in the frequency range of 20 × 20 ppm; Figure S7: ^1^H-^1^H TOCSY spectrum (mixing time parameter—100 ms) of FFA in DMSO-d_6_ was recorded on a Bruker Avance III spectrometer, 500 MHz, in the frequency range of 20 × 20 ppm; Figure S8: Results of quantum chemical calculations demonstrating barriers of intramolecular rotation associated with conformers A(B) and C(D) along the O=C_1_-C_13_-C_6_ angle in the FFA molecule; Table S1: Chemical shifts in the 1D NMR spectra and intramolecular interactions determined from the 2D spectra of the FFA molecule in DMSO; Table S2: Dihedral angles τ_1_[C_2_-N-C_3_-C_7_] and respective molecular structures comprising eight polymorphic forms of FFA; Table S3: Normalized integral intensities of the cross-peaks, cross-relaxation rates and inter-proton distances in the FFA molecule used to calculate the conformer populations in DMSO-d_6_; Table S4: Normalized integral intensities of the cross-peaks, cross-relaxation rates and inter-proton distances in the FFA molecule used to calculate the conformer populations in SC-CO_2_+DMSO-d_6_.
