# Supplementary material for: Investigation of the Spatial Structure of Flufenamic Acid in Supercritical Carbon Dioxide Media via 2D NOESY
Source: Materials (Basel). 2023 Feb 11;16(4):1524. doi: 10.3390/ma16041524 (PMC9961892; doi:10.3390/ma16041524)
Supplement: Supplementary file 1 [file materials-16-01524-s001.zip › materials-2146985-supplementary.pdf]

Supplementary Materials

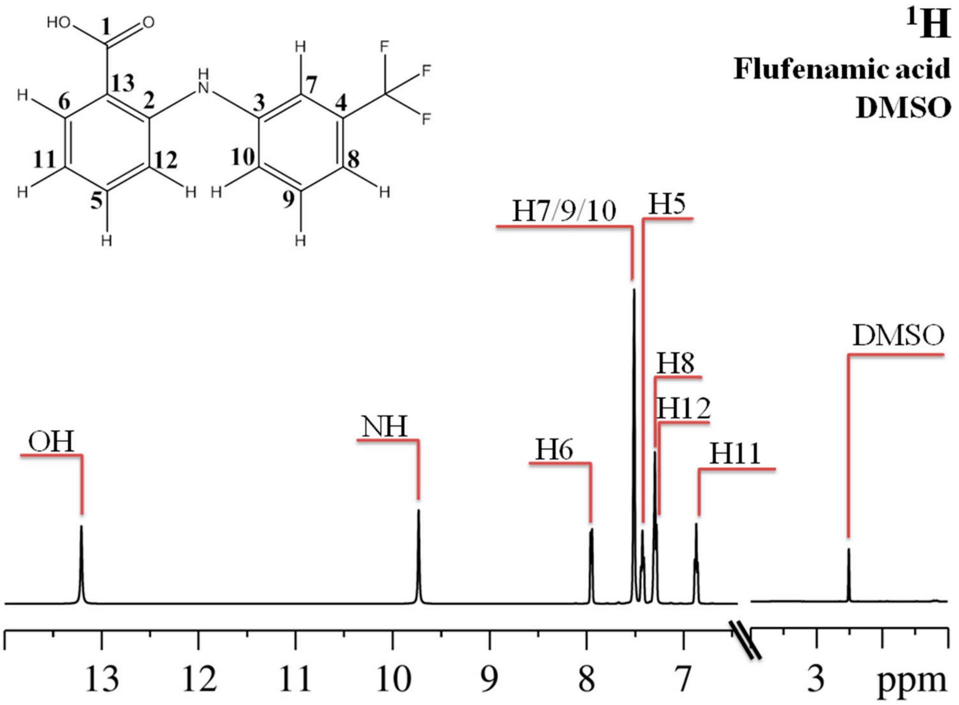

Figure S1. ^{1}H NMR spectrum of FFA in DMSO-d_6 was recorded on a Bruker Avance III spectrometer, 500 MHz, in the frequency range of 20 ppm.

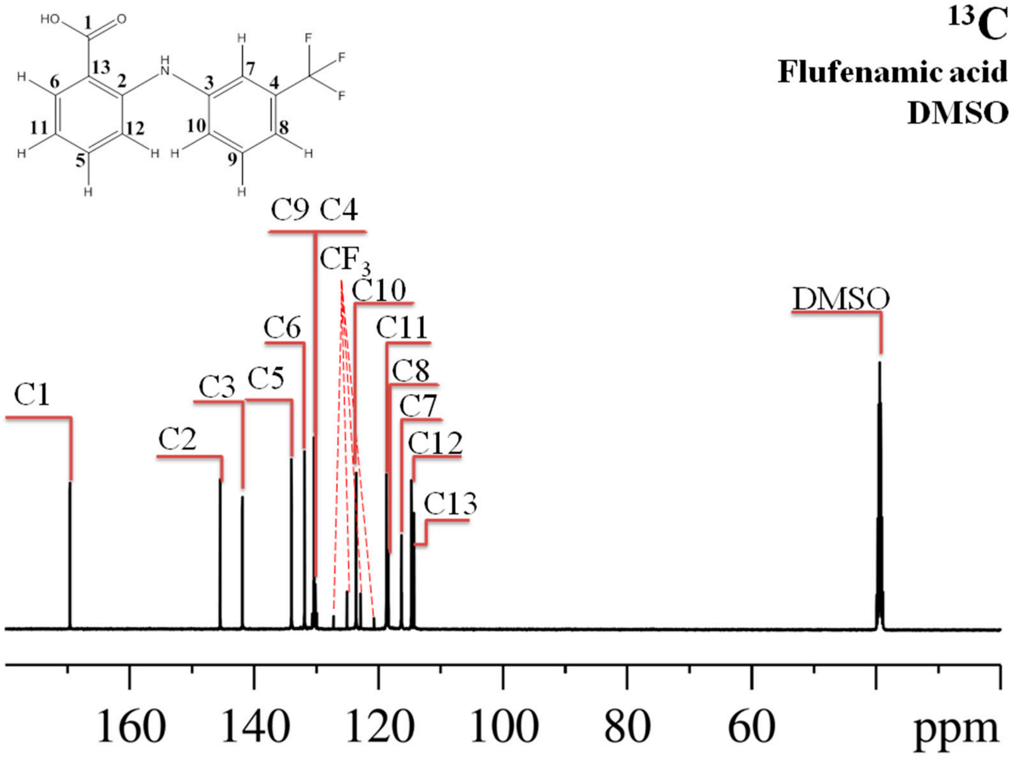

Figure S2. ^{13}C NMR spectrum of FFA in DMSO-d_6 was recorded on a Bruker Avance III spectrometer, 500 MHz, in the frequency range of 276 ppm.

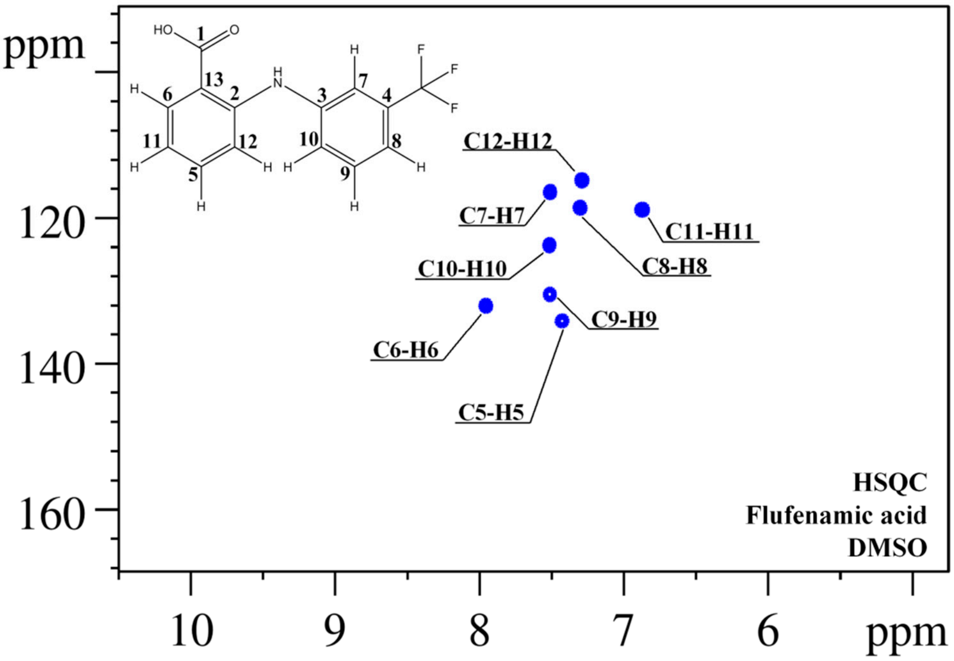

Figure S3. ^{1}H-^{13}C HSQC spectrum of FFA in DMSO-d_6 was recorded on a Bruker Avance III spectrometer, 500 MHz, in the frequency range of 20 × 276 ppm.

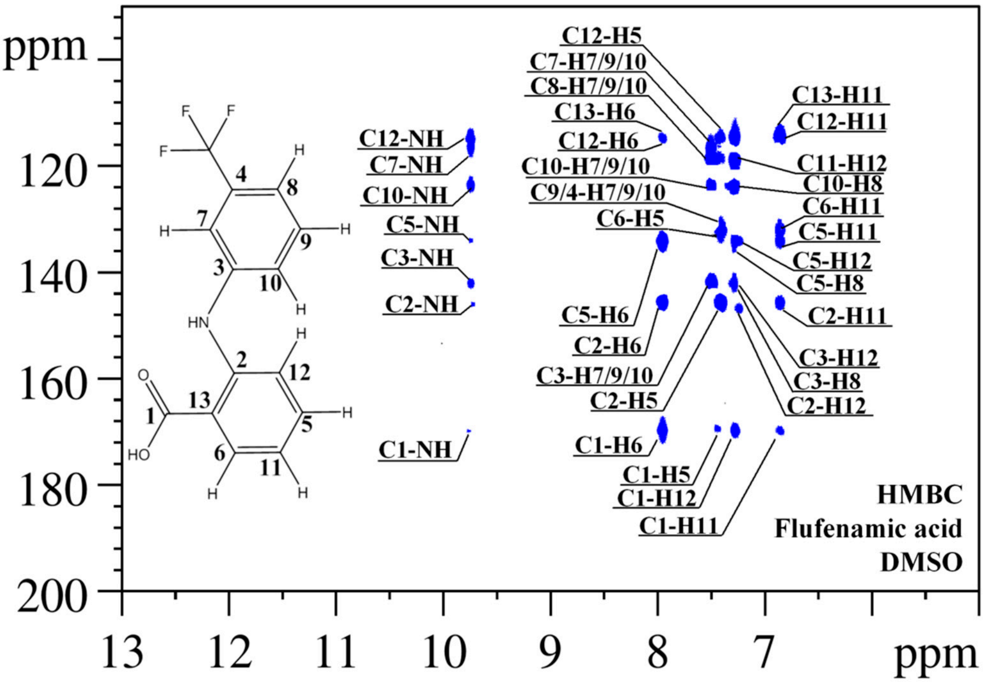

Figure S4. ^{1}H-^{13}C HMBC spectrum of FFA in DMSO-d_6 was recorded on a Bruker Avance III spectrometer, 500 MHz, in the frequency range of 20 × 276 ppm.

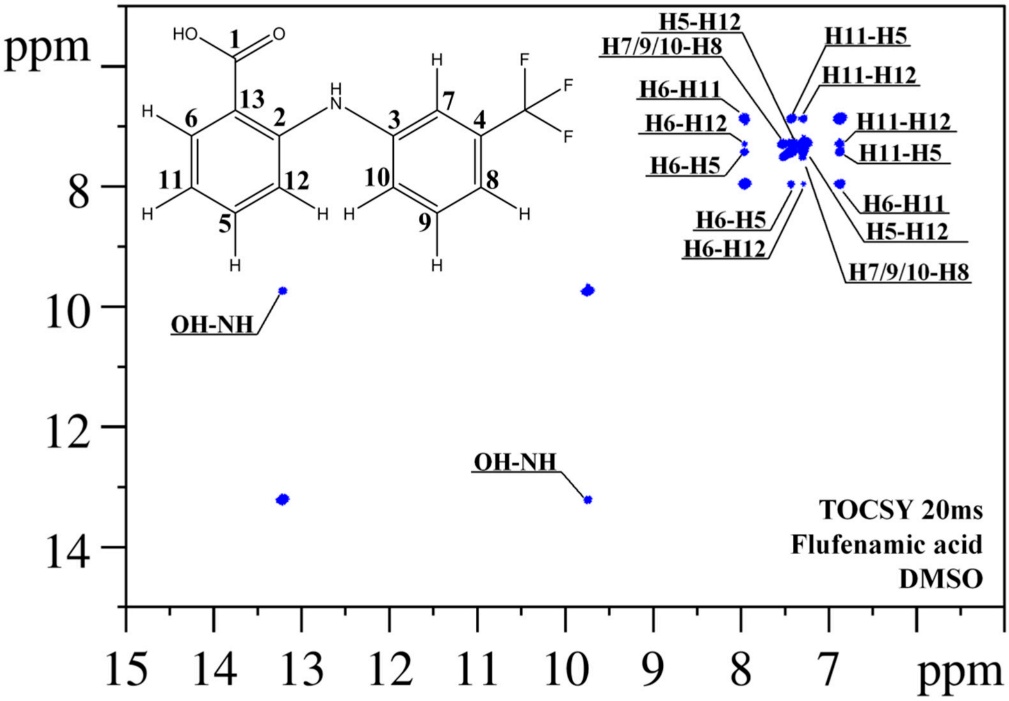

Figure S5. ^{1}H-^{1}H TOCSY spectrum (mixing time parameter—20 ms) of FFA in DMSO-d_6 was recorded on a Bruker Avance III spectrometer, 500 MHz, in the frequency range of 20 × 20 ppm.

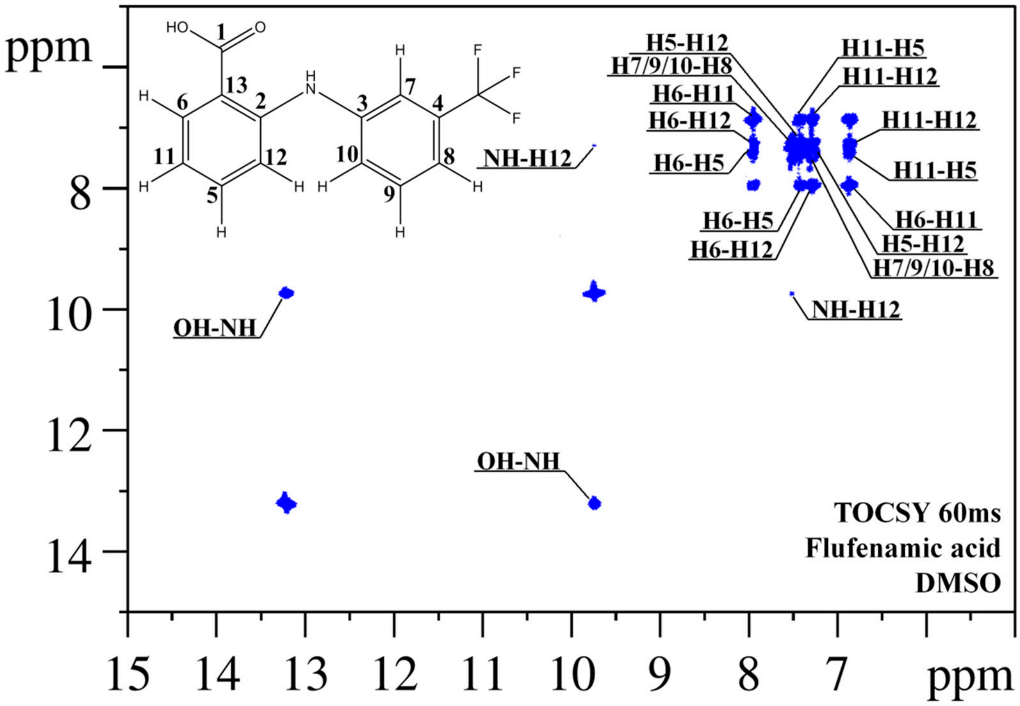

Figure S6. ^{1}H-^{1}H TOCSY spectrum (mixing time parameter—60 ms) of FFA in DMSO-d_6 was recorded on a Bruker Avance III spectrometer, 500 MHz, in the frequency range of 20 × 20 ppm.

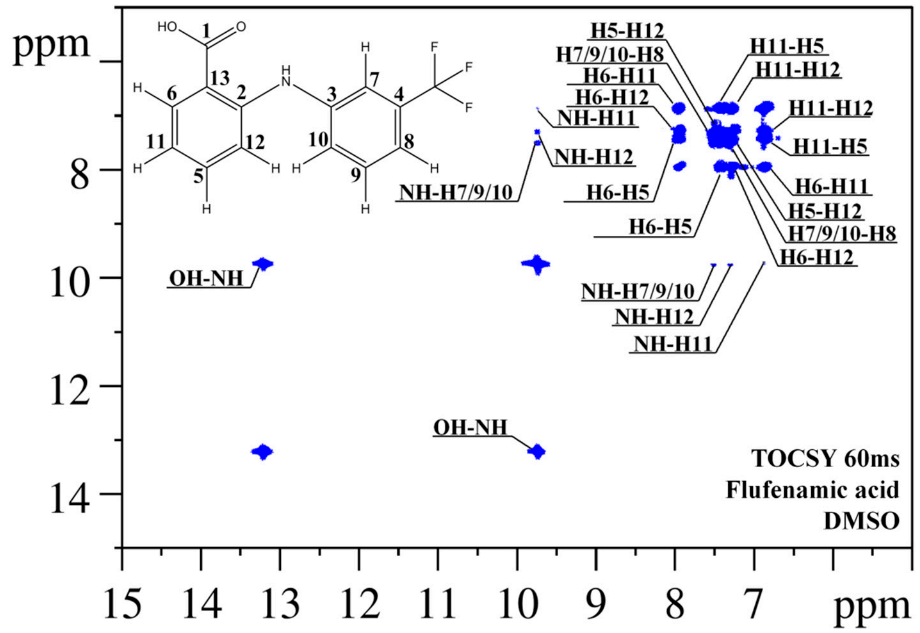

Figure S7. ^{1}H-^{1}H TOCSY spectrum (mixing time parameter—100 ms) of FFA in DMSO-d_6 was recorded on a Bruker Avance III spectrometer, 500 MHz, in the frequency range of 20 × 20 ppm.

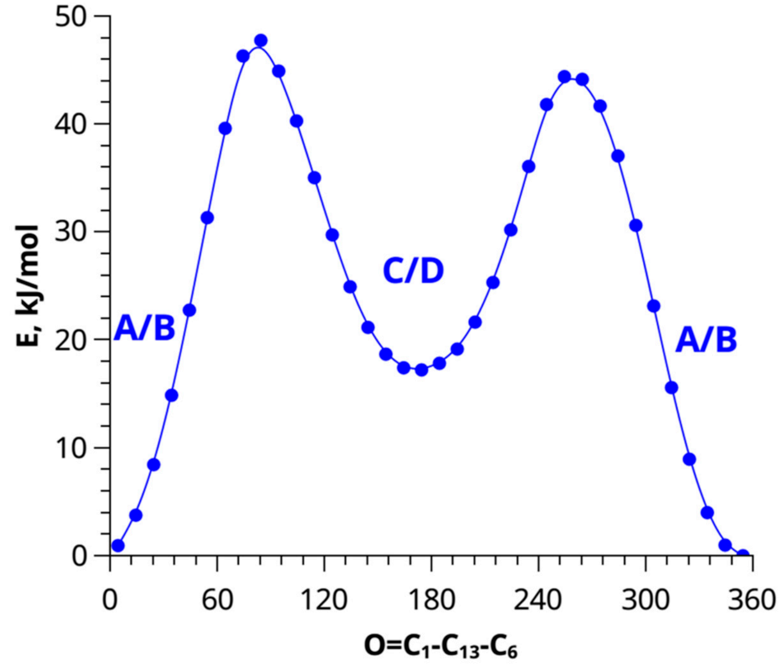

Figure S8. Results of quantum chemical calculations demonstrating barriers of intramolecular rotation associated with conformers A(B) and C(D) along the $O{=}C_1$-C_{13}-C_6 angle in the FFA molecule.

Table S1. Chemical shifts in the 1D NMR spectra and intramolecular interactions determined from the 2D spectra of the FFA molecule in DMSO.

^{13}C	δ ^{13}C, ppm	^{1}H	δ ^{1}H, ppm	HSQC	HMBC	TOCSY			NOESY
						20 ms	60 ms	100ms	
C1	169.62	OH	13.21	-	C1-NH<br>C1-H6<br>C1-H5<br>C1-H12<br>C1-H11	OH-NH	OH-NH	OH-NH	OH-NH<br>OH-H6<br>OH-H7/9/10
C2	145.47	-	-	-	C2-NH<br>C2-H6<br>C2-H5<br>C2-H11<br>C2-H12	-	-	-	-
C3	141.89	-	-	-	C3-NH<br>C3-H7/9/10<br>C3-H8<br>C3-H12	-	-	-	-
C5	133.99	H5	7.43	C5-H5	C5-NH<br>C5-H6<br>C5-H8<br>C5-H11<br>C5-H12	H5-H12	H5-H12	H5-H12	H5-H11
C6	131.90	H6	7.95	C6-H6	C6-H5<br>C6-H11	H6-H5<br>H6-H12<br>H6-H11	H6-H5<br>H6-H12<br>H6-H11	H6-H5<br>H6-H12<br>H6-H11	H6-H11
C7	116.33	H7/9/10	7.51	C7-H7	C7-NH<br>C7-H7/9/10	H7/9/10-H8	H7/9/10-H8	H7/9/10-H8	H7/9/10-<br>H8/12
C9	132.34			C9-H9	C9/4-H7/9/10				
C10	122.61			C10-H10	C10-NH<br>C10-H7/9/10<br>C10-H8				
C4	130.26	-	-	-	-	-	-	-	-
CF_3	127.24<br>125.09<br>122.91<br>120.74	-	-	-	-	-	-	-	-
C11	118.73	H11	6.87	C11-H11	C11-H12	H11-H5<br>H11-H12	H11-H5<br>H11-H12	H11-H5<br>H11-H12	-
C8	118.46	H8	7.30	C8-H8	C8-H7/9/10	-	-	-	-
C12	114.71	H12	7.28	C12-H12	C12-NH<br>C12-H5<br>C12-H6<br>C12-H11	-	-	-	-
C13	114.31	-	-	-	C13-H6<br>C13-H5<br>C13-H11<br>C13-H12	-	-	-	-
-	-	NH	9.73	-	-	-	NH-H12	NH-H11<br>NH-H12<br>NH-H7/9/10	NH-H6<br>NH-H7/9/10

Table S2. Dihedral angles τ1[C2-N-C3-C7] and respective molecular structures comprising eight polymorphic forms of FFA.

Polymorph	τ1[C2-N-C3-C7], °	Conformer Structure
FFA I	−131.71	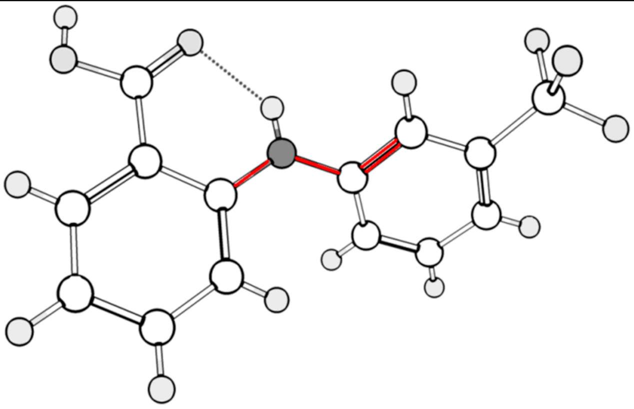
FFA II	141.30	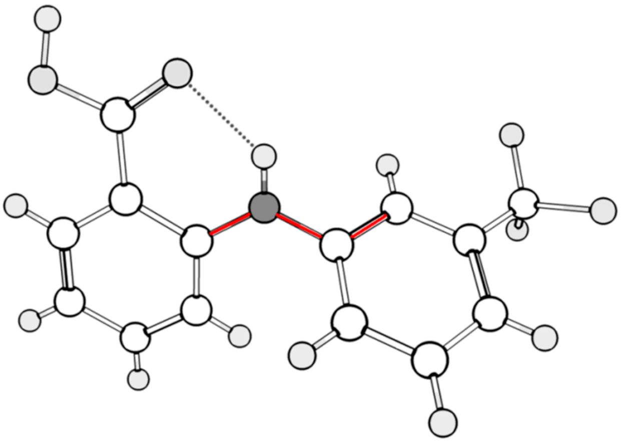
FFA III	−8.35	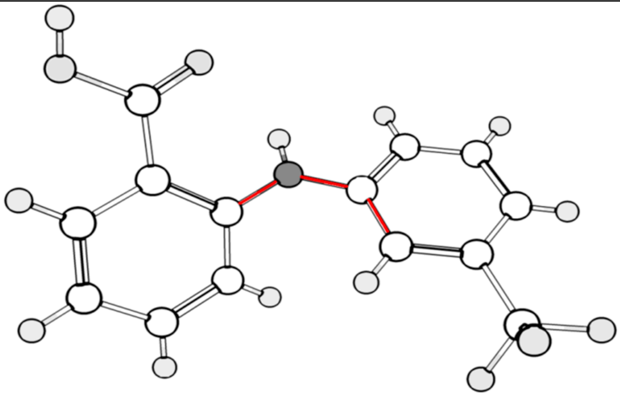
FFA IV	147.52 [a]<br>−142.67 [b]	

	28.84 [c]	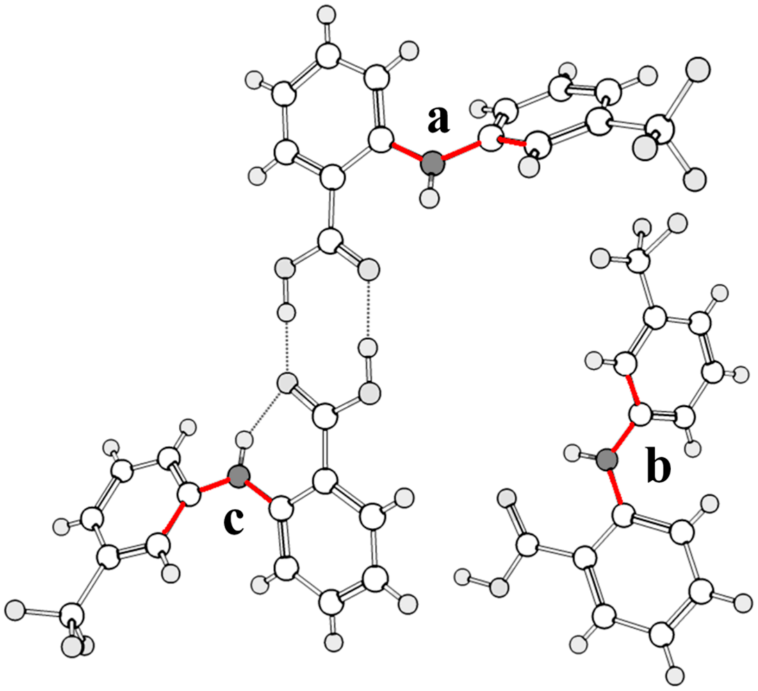
FFA V	155.85 [a]	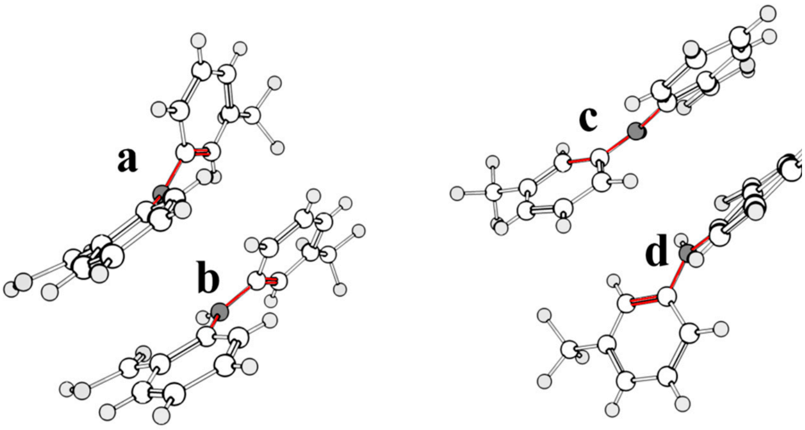
	143.15 [b]	
	145.18 [c]	
	153.91 [d]	
FFA VI	24.50 [a]	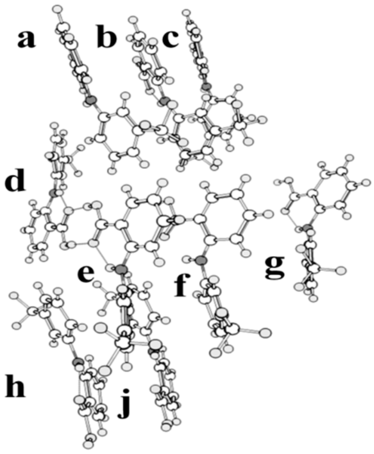
	145.84 [b]	
	−147.42 [c]	
	−27.56 [d]	
	140.70 [e]	
	−148.63 [f]	
FFA VII	−43.34 [a]	

	41.66 [b]	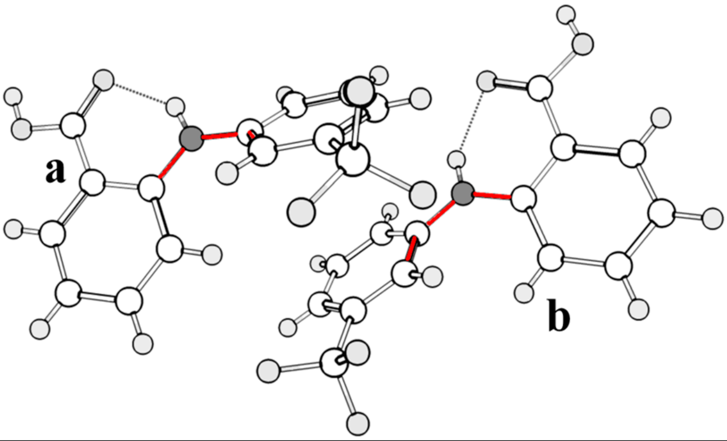
FFA VIII	−25.31 [a]	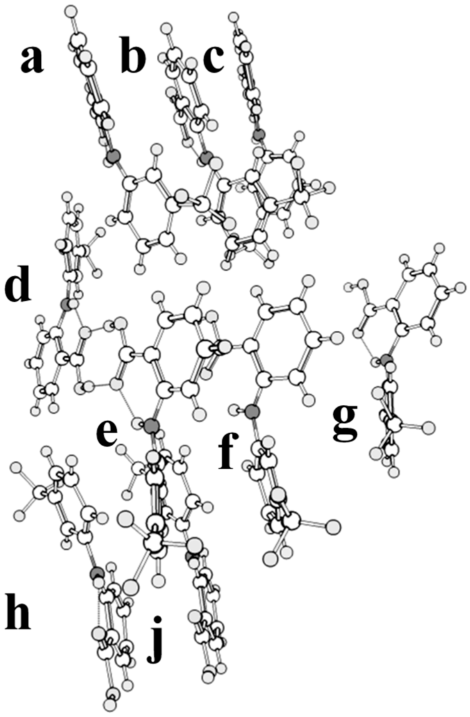
	−38.26 [b]	
	143.97 [c]	
	−32.55 [d]	
	40.95 [e]	
	21.12 [f]	
	35.29 [g]	
	27.43 [h]	
	28.86 [j]	

Table S3. Normalized integral intensities of the cross-peaks, cross-relaxation rates and inter-proton distances in the FFA molecule used to calculate the conformer populations in DMSO-d_6.

Groups	r_{calc}, Å	Dist.	Mixing Time	Relative Integral Intensity	Cross-Relaxation Rate, s^{-1}	r_{exp}, Å
A-D	2.47	H6-H11 (Ref)	0.05	0.0037	$3.89 \pm 0.14 \times 10^{-2}$	-
			0.10	0.0057		
			0.15	0.0076		
			0.20	0.0092		
			0.25	0.0118		
			0.30	0.0144		
			0.35	0.0152		
			0.40	0.0166		
			0.45	0.0207		
			0.50	0.0216		
			0.55	0.0246		
			0.60	0.0247		
			0.65	0.0269		
			0.70	0.028		
			0.75	0.0317		
			0.80	0.0329		
A+C	2.48	NH-H7	0.05	0.0017	$2.05 \pm 0.05 \times 10^{-2}$	2.75 ± 0.03
			0.10	0.003		
			0.15	0.0047		
			0.20	0.0058		
			0.25	0.0067		
			0.30	0.0078		
			0.35	0.0085		
			0.40	0.0093		
B+D	3.58		0.45	0.0104		
			0.50	0.0111		
			0.55	0.0116		
			0.60	0.0131		
			0.65	0.0139		
			0.70	0.0158		
			0.75	0.0171		
			0.80	0.0181		

Table S4. Normalized integral intensities of the cross-peaks, cross-relaxation rates and inter-proton distances in the FFA molecule used to calculate the conformer populations in SC-CO_2+DMSO-d_6.

Groups	r_{calc}, Å	Dist.	Mixing Time	Relative Integral Intensity	Cross-Relaxation rate, s^{-1}	r_{exp}, Å
A-D	2.47	H6-H11 (Ref)	0.10	0.0048	$3.89 \pm 0.14 \times 10^{-2}$	-
			0.20	0.0103		
			0.30	0.0097		
			0.40	0.013		
			0.50	0.0211		
			0.60	0.0233		
			0.70	0.0252		
			0.80	0.0306		
			0.90	0.0327		
A+C	2.48	NH-H7	0.10	0.0026	$2.05 \pm 0.05 \times 10^{-2}$	2.75 ± 0.03
			0.20	0.0031		
			0.30	0.0042		
			0.40	0.0044		
			0.50	0.005		
			0.60	0.0062		
			0.70	0.0074		
			0.80	0.0088		
B+D	3.58		0.90	0.0099		
